# Ways to Enhance Lymphocyte Trafficking into Tumors and Fitness of Tumor Infiltrating Lymphocytes

**DOI:** 10.3389/fonc.2013.00231

**Published:** 2013-09-11

**Authors:** Matteo Bellone, Arianna Calcinotto

**Affiliations:** ^1^Cellular Immunology Unit, Department of Immunology, Infectious Diseases and Transplantation, San Raffaele Scientific Institute, Milan, Italy; ^2^Università Vita Salute San Raffaele, Milan, Italy

**Keywords:** cytotoxic T lymphocyte, vaccine, adoptive T cell therapy, pH, redox, proton pump inhibitor, NGR-TNF, combination therapy

## Abstract

The tumor is a hostile microenvironment for T lymphocytes. Indeed, irregular blood flow, and endothelial cell (EC) anergy that characterize most solid tumors hamper leukocyte adhesion, extravasation, and infiltration. In addition, hypoxia and reprograming of energy metabolism within cancer cells transform the tumor mass in a harsh environment that limits survival and effector functions of T cells, regardless of being induced *in vivo* by vaccination or adoptively transferred. In this review, we will summarize on recent advances in our understanding of the characteristics of tumor-associated neo-angiogenic vessels as well as of the tumor metabolism that may impact on T cell trafficking and fitness of tumor infiltrating lymphocytes. In particular, we will focus on how advances in knowledge of the characteristics of tumor ECs have enabled identifying strategies to normalize the tumor-vasculature and/or overcome EC anergy, thus increasing leukocyte-vessel wall interactions and lymphocyte infiltration in tumors. We will also focus on drugs acting on cells and their released molecules to transiently render the tumor microenvironment more suitable for tumor infiltrating T lymphocytes, thus increasing the therapeutic effectiveness of both active and adoptive immunotherapies.

## Introduction

Active and adoptive cancer immunotherapies have breached the wall between bench and bedside at last, and have just entered a new golden age. This is the result of several concomitant technological advancements and breakthrough discoveries. On the one hand, powerful technical tools (e.g., tetramers and live imaging) have been made available to more deeply investigate the interactions between the growing tumor and the host, and especially the immune system. Thus, sophisticated genetically engineered animal models have allowed building new theories on the process of cancer immune surveillance (1). Furthermore, high-throughput technologies are making possible investigating the tumor microenvironment as a whole (2), and genomic landscaping, proteomic profiling, and more recently metabolomics and algorithms applied to cancer histochemistry (3–8) are drawing an entirely new picture of the tumor mass. On the other hand, strong efforts from hundreds of laboratories around the word in the last 20 years have defined tumor-associated antigens (TAAs) and adjuvants to such an high degree of knowledge [e.g.,(9)] that active immunotherapy has eventually reached the bedside with the first FDA approved cancer vaccine for metastatic prostate cancer patients (10). Even more importantly, clinical grade *in vitro* expanded tumor infiltrating lymphocytes (TILs) and genetically engineered T cells have demonstrated the full potential of adoptive immunotherapy (11, 12).

**Figure 1:**
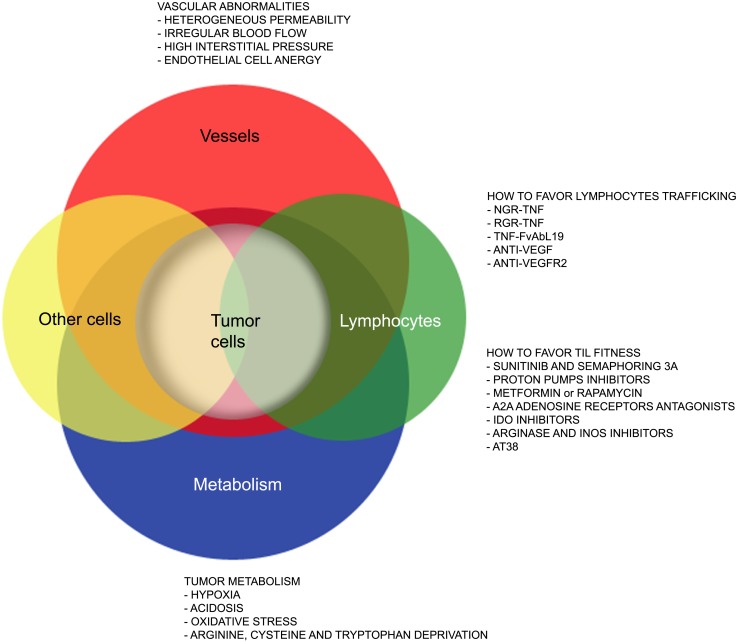
**Strategies that favor lymphocyte trafficking into tumors and fitness of TILs**. The cartoon highlights abnormalities of tumor-associated vessels and alterations of the metabolism within the tumor microenvironment that limit lymphocyte trafficking into tumor and TIL anti-tumor activities. Strategies to overcome such hurdles are also indicated.

Yet, several hurdles still need to be overcome (Figure 1) to extend such treatments to the majority of cancer patients. Firstly, the tumor mass is characterized by abnormal tumor vessels and interstitium that limit leukocyte adhesion, extravasation, and infiltration (13), and favors hypoxia and reprograming of energy metabolism within cancer cells (14). Metabolic alterations within the tumor mass also limit T cell functions, and the tumor microenvironment eventually becomes a site of immune privilege where several cancer cell intrinsic and extrinsic mechanisms suppress the tumor-specific T cell response (15).

Here, we will summarize on recent advances in our understanding of the characteristics of tumor-associated neo-angiogenic vessels as well as of the tumor metabolism that may impact on T cell trafficking and fitness of TILs. We will also report on drugs acting on cells and their released molecules to transiently render the tumor microenvironment more suitable for tumor TILs (Figure 1), thus increasing T cell trafficking into tumors and the therapeutic effectiveness of both active and adoptive immunotherapies.

## T Cell Adhesion to the Endothelium, Extravasation, and Infiltration within Inflamed Tissues

Once a T cell has been activated in secondary lymphoid organs, it reaches the blood flow and navigates within vessels to the site of extravasation, which usually coincides with a site of inflammation. Activated T cells prefer to exit the blood stream at the level of post-capillary venules, where the hemodynamic shear stress is lower than in arteries and capillaries and the endothelium is more prone to extravasation. Activated T cells travel more efficiently than naïve T cells to inflamed tissues because they upregulate adhesion molecules and chemoattractant receptors for inflammation induced ligands. Transendothelial migration involves specific adhesive interactions between T cells and endothelial cells (ECs) that guide the lymphocytes from the vascular compartment to the extravascular tissue. We refer the interested reader to excellent reviews on this topic (16–20). In brief, T cells undergo four distinct adhesion steps during their migration through blood vessels. These include tethering, rolling, activation, and arrest. Tethering and rolling of leukocytes are mediated by interactions between selectins and specific carbohydrate moieties bound to a protein backbone (21), which allow rapid engagement with high tensile strength. The selectins are a family of three C-type lectins expressed by bone marrow-derived cells and ECs. l-selectin (CD62L) is expressed by all myeloid cells, naïve T cells, and some activated and memory cells. P-selectin (CD62P) is found in secretory granules of platelets and ECs and is expressed on the cell surface after activation by inflammatory stimuli. E-selectin (CD62E) is expressed by acutely inflamed ECs in most organs and by non-inflamed skin microvessels. Thus, P-selectin glycoprotein ligand 1 (PSGL-1) and CD43 on activated T cells engage CD62P and CD62E on activated ECs, respectively. Rolling T cells receive signals from chemokines on ECs, which induce modulation of integrins to acquire high avidity for their ligands. Integrins may participate to the rolling phase but are essential for the firm adhesion of leukocytes. In particular, activated T cells depend on lymphocyte function-associated antigen 1 (LFA-1), very late antigen-4 (VLA-4; α4β1), and α4β7 for their interactions with activated ECs that express intracellular adhesion molecule 1 (ICAM-1), intracellular adhesion molecule 2 (ICAM-2), VCAM-1, and mucosal addressin-cell adhesion molecule type 1 (MAdCAM-1), respectively (22).

Quiescent ECs poorly interact with circulating leukocytes. Autacoid mediators released by mast cells and other cells of the innate immunity, upon stimulation by inflammatory signals (e.g., infection and tissue damage), cause rapid enhancement of venular permeability, translocation of integrins, and chemokines from intracellular stores to the cell surface and formation of a provisional matrix (23), all processes that favor T cell-EC interactions.

A very different scenario may characterize T cell-EC interactions in tumor-associated vessels.

## Tumor-Associated Modifications of the Endothelium Hamper T Cell Adhesion, Extravasation, and Tumor Infiltration

While acute inflammation is an efficacious means by which the organism repairs a tissue that has been damaged by a physical insult or infection, chronic inflammation has emerged has indispensable requisite for chronic diseases including cancer (24). Indeed, tumor-promoting inflammation has been recently recognized has as an enabling characteristic that allows cancer cells to acquire multiple hallmark capabilities including sustaining proliferative signaling, resisting cell death, avoiding immune destruction, activating invasion, and metastasis and inducing angiogenesis (25). Virtually any neoplastic lesion contains immune inflammatory cells although with variable densities (26). Yet, gene expression profiling of the total cellular composition of tumors has evidenced at least two subsets of tumors. The first “inflamed” subset is characterized by transcripts encoding innate immune cell molecules, chemokines that can contribute to effector T cell recruitment, various T lineage-specific markers, and, paradoxically, immune inhibitory mechanisms. Conversely, the “not-inflamed” phenotype is distinguished for high expression of angiogenesis-associated factors as well as macrophages and fibroblasts (2). Thus, it has been hypothesized that TILs effectively extravasate in inflamed tumors but are inhibited by immunosuppressive mechanisms, including indoleamine-2,3-dioxygenase (IDO), programed cell death 1 ligand 1 (PD-L1), and forkhead box P3 (FoxP3)^+^ regulatory T cells (Tregs), whereas, T cell migration is defective in not-inflamed tumors (2).

**Figure 2:**
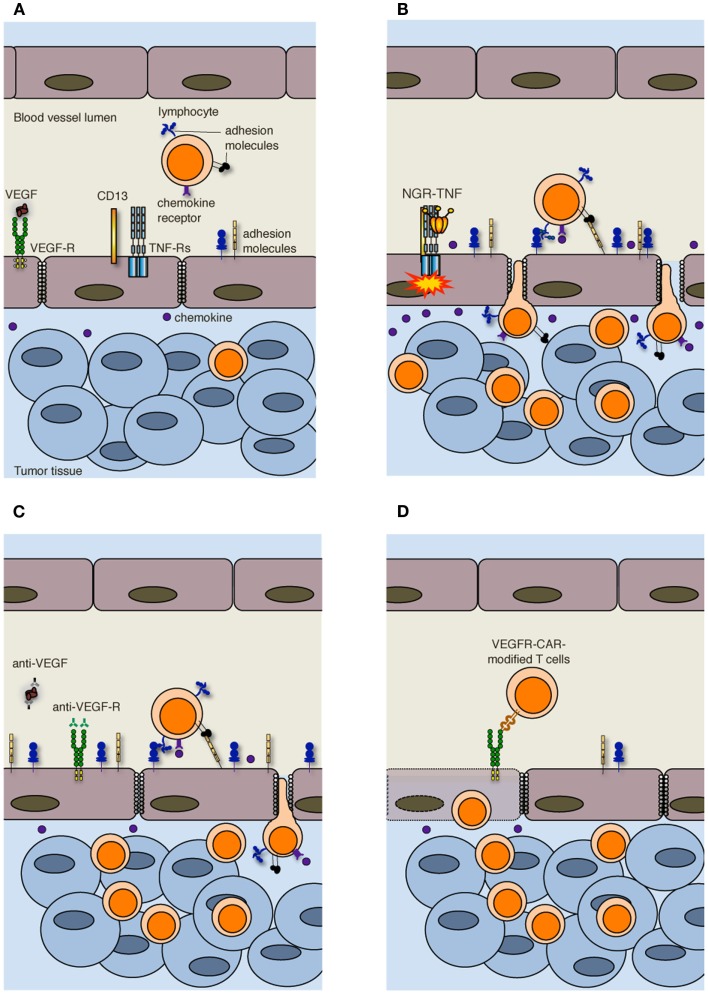
**Strategies to increase T cell infiltration into tumors**. **(A)** Increased interstitial pressure, heterogeneous permeability and irregular blood flow, together with reduced expression of adhesion molecules on ECs, limit lymphocyte penetration in tumors. **(B)** NGR-TNF, which selectively binds CD13 expressed in ECs of neo-angiogenic vessels and favors the interaction of TNF with TNF receptors (TNF-Rs), alters tumor vessel permeability by loosening VE-cadherin dependent adherence junctions, induces up-regulation of adhesion molecules in ECs, and elicits the release of pro-inflammatory cytokines and chemokines, thereby favoring the recruitment and extravasation of T lymphocytes. **(C)** Anti-VEGF and anti-VEGF-R antibodies both transiently normalize the tumor-vasculature and overcome EC anergy, thus favoring T cell trafficking within tumors. **(D)** Also immunization against VEGF-R2 or the adoptive transfer of autologous T cells genetically engineered to express chimeric antigen receptor targeted against VEGF-R2 (VEGFR-CAR) favor tumor infiltration by T cells, although the mechanism has not yet been clarified. It has been proposed that VEGF-R-specific T cells kill both ECs and MDSCs and Tregs (not shown) that express VEGF-R.

More in general, vessels are irregularly distributed within the tumor mass that, when reaches 1–2 mm in diameter, presents a patchy distribution of less-perfused and hypoxic areas (27). Hypoxia is one of the strongest stimulators of angiogenesis, largely through the expression of hypoxia inducible transcription factors [HIFs; (28)]. Tumor vessels that sprout from existing ones are disorganized, tortuous, dilated, saccular, and leaker then the normal ones. Also the composition of the vessel is abnormal, and ECs may acquire aberrant morphology, pericytes may be absent or loosely attached, and the vessel may lack basement membrane or have it unusually thick (13, 29). In addition, tumor cells may mimic ECs and generate vascular conduits, which however, are even more abnormal (30). All together, these vascular abnormalities render tumor vessels leaker then normal ones, may increase the interstitial pressure, cause heterogeneous permeability, and promote irregular blood flow, therefore making leukocyte trafficking within the tumor mass difficult. Interstitial pressure is also increased by the extrinsic compression of tumor vessels by proliferating cancer cells. In addition, angiogenic factors such as vascular EC growth factors (VEGFs) and fibroblast growth factors (FGFs) cause down-regulation of ICAM-1/2, VCAM-1, and CD34 on ECs, a phenomenon defined as “EC anergy” (31–33). Thus, the few effector T cells that circulate in tumor vessels, regardless of being induced *in vivo* by vaccination or adoptively transferred (34, 35), can hardly interact with ECs and begin their migration through blood vessels (Figure 2A). In line with this view, gene expression profiling and *in situ* immunohistochemical staining of large cohorts of cancer patients have shown that more aggressive tumors are characterized by peritumoral immune infiltrates (36), whereas a strong *in situ* accumulation of T cells both in the center of the tumor and the invading margin correlates with a favorable prognosis regardless of the local extent of the tumor and of metastasis (37).

## Ways to Favor T Cell Adhesion to Tumor-Associated Endothelial Cells, Extravasation, and Tumor Infiltration

Crossing the abnormal tumor vessel barrier and interstitium is one major hurdle for tumor-specific T cells that have reached the tumor mass (Figure 1). Few years ago, we proposed that delivery of vasoactive inflammatory cytokines like tumor necrosis factor α (TNF) to neo-angiogenic vessels might represent a good strategy to induce selective activation of ECs in tumor tissues, thereby enhancing T cell extravasation and tumor infiltration (38). TNF is produced in the tumor microenvironment mainly by macrophages, but also by smooth muscle cells, ECs and tumor cells (39), and it affects primarily the tumor-associated vasculature (40). Indeed, most tumor cells and vessels of normal tissues are resistant to TNF (41). Depending on the amount of TNF that reaches the tumor mass, its effects range from EC activation, to increased vessel permeability, EC damage, and massive hemorrhagic necrosis (42). The *in vivo* effects of TNF have been well characterized both in pre-clinical models and in humans undergoing isolated limb perfusion, a regional cancer therapy used to deliver high doses of a drug into the bloodstream of a limb avoiding severe systemic side effects (42). An alternative strategy to avoid TNF-induced systemic toxicity is indeed to selectively target minute amounts of the cytokine to the tumor vessels. Selective delivery of TNF to tumor vessels has been achieved by fusing this cytokine with a tumor-vasculature-homing peptide that contains the Cys-Asn-Gly-Arg-Cys (NGR) sequence, a ligand of a CD13 isoform expressed by neo-angiogenic vessels (43, 44). The new moiety called NGR-TNF was shown to transiently enhance tumor vessel permeability (45), thus increasing the penetration of chemotherapy agents in murine models of lymphoma, melanoma, and spontaneous prostate cancer without TNF-related systemic toxicity (46, 47). NGR-TNF is currently under clinical investigation in various clinical studies in cancer patients (48).

In accordance with our original hypothesis (38), we have recently shown that extremely low doses of NGR-TNF (5 ng/Kg) are sufficient to induce the up-regulation of VCAM-1 and ICAM-2 on the endothelial lining of tumor vessels as well as the release, in the tumor microenvironment, of chemokines that favor T-cell trafficking (Figure 2B). Rapid and transient modification of the tumor microenvironment can enhance the infiltration of either fully activated endogenous or adoptively transferred T cells in transplantable melanoma and autochthonous prostate cancer (49). Additionally, we have demonstrated that NGR-TNF can increase the therapeutic efficacy of tumor vaccines and adoptive immunotherapy with no evidence of toxic reactions (49). The effects of NGR-TNF on tumor infiltration by leukocytes go beyond the transient activation of tumor-associated ECs (50). Indeed, NGR-TNF transiently modifies the endothelial barrier function by loosening VE-cadherin dependent adherence junctions (51), thus favoring T cell extravasation (Figure 2B). It can also transiently reduce hypoxic areas of the tumor (52) and favor TIL proliferation and survival (50).

A similar compound, consisting of TNF fused to another tumor-vasculature-homing peptide (RGR) has been recently shown to stabilize tumor vessels and to enhance active immunotherapy in experimental pancreatic neuroendocrine tumors (52). Notably, a recent phase II study of NGR-TNF (0.8 μg/m2) in combination with doxorubicin in relapsed ovarian cancer patients showed that patients with baseline peripheral blood lymphocyte count higher than the first quartile had improved progression-free survival and overall survival (53), therefore suggesting that a similar effect may occur in humans.

Other strategies have been pursed to target TNF to the tumor. As an example, TNF has been fused with the single chain Fv Ab L19, which is specific for the extradomain B of fibronectin expressed by the tumor neovasculature (54). However, the location of the target molecules in tumor vessels and their level of expression are different from that of CD13, and additional studies are necessary to investigate whether this compound acts in synergy with active or adoptive immunotherapy.

Leukocyte infiltration in tumors can also be favored by the use of classic anti-angiogenic drugs. VEGF is the focus of most of these approaches (55). The importance of VEGF-mediated mechanisms in cancer is underlined by clinical data showing that the expression of VEGF in tumor tissue is negatively correlated with the presence of TILs. This was reported to be one of the strongest prognostic factors in ovarian carcinoma (56). In addition, VEGF negatively regulates functional maturation of and antigen presentation by dendritic cells (DCs), favors the accrual and activity of cell populations with immunosuppressive functions including myeloid derived suppressor cells [MDSCs; (57)] and regulatory T cells [Tregs; (58)], and induces T cell apoptosis, therefore contributing to the immunosuppressive tumor microenvironment (59). Over the last decades several therapeutic approaches have been proposed to counteract VEGF and neoangiogenesis, such as anti-VEGF antibodies and tyrosine kinase inhibitors of multiple pro-angiogenic growth factor receptors (13). Inhibition of VEGF interaction with its receptors has been also reported to be at the basis of vessel “normalization” (29). Anti-angiogenic drugs transiently normalize the tumor-vasculature, pruning away immature and leaky vessels and remodeling the remaining vasculature. As a result, the enhanced oncotic pressure gradient together with decreased interstitial fluid pressure and hydrostatic pressure gradient facilitate delivery of oxygen, nutrients, and also chemotherapeutic agents into the tumor microenvironment (13). Some of these strategies can also overcome EC anergy and promote leukocyte infiltration in tumors [Figure 2C; (60–62)]. In addition, it has been reported that lower-dose of anti-VEGF (DC101; 10 mg/Kg), when compared with the standard high dose (40 mg/Kg), normalizes the tumor-vasculature, favors extravasation of T cells, reduces the fraction of MDSCs, and polarizes macrophages toward an M1 phenotype within the tumor mass (63). Thus, anti-angiogenic drugs and TNF targeting are conceptually different approaches, as the former aims at vessel normalization, whereas the latter exploits the cytokine as an inflammatory agent that induces vascular activation.

Alternative approaches to target the VEGF-VEGF receptor (VEGFR) pathway are immunization against VEGFR-2 (64) or the adoptive transfer of autologous T cells genetically engineered to express a chimeric antigen receptor targeted against VEGFR-2 [Figure 2D; (65)]. The simultaneous targeting of VEGFR-2 and TAAs by a mixture of genetically engineered T cells expressing a chimeric antigen receptor targeting VEGFR-2 and T cells expressing a TCR specific for a melanoma-associated TAA synergistically eradicated established melanoma tumors in mice and prolonged their tumor free survival (66). The mechanism behind this synergy is still under investigation, and the transduction of anti-VEGFR-2 CAR into TCR transgenic T cells did not enhance the therapeutic efficacy of adoptively transferred cells (66). Because of the extensive tumor necrosis induced by the adoptive transfer of T cells, vessels could not be investigated in these tumors (66). The authors favor the hypothesis that anti-VEGFR-2 T cells not only target ECs but also suppressor cell populations including MDSCs and Tregs that express VEGFR-2 (67, 68).

In general, anti-VEGF-mediated transient normalization of tumor vessels lasts between few days to a month (29). Unfortunately, the anti-angiogenic drugs available to date are not sufficiently selective in damaging only neo-angiogenic vessels. Risks of sustained and/or aggressive anti-angiogenic therapies are the unselected recruitment of pro-angiogenic inflammatory cells, and excessive trimming of vessels with inadequate delivery of oxygen and drugs. The latter effect may be dangerous also for highly vascularized tissues, including the cardiovascular, endocrine, and nervous systems (69).

As mentioned before, targeting TNF to the tumor vessels enhances tumor permeability to chemotherapeutic agents (48). We have recently reported that the combination of active or adoptive immunotherapy, vascular targeting, and chemotherapy act in synergy against melanoma (49). Our preliminary results also suggest that in the context of adoptive T cell therapy after hematopoietic stem cell transplantation (70, 71), NGR-TNF dramatically increases the infiltration of TILs into the prostate of mice affected by autochthonous prostate cancer (49) and contributes to tumor debulking (Mondino A. Personal communication).

Interestingly, chemotherapeutic agents, beside their effects in promoting anti-tumor immunity by inducing a more immunogenic death of cancer cells, increasing their sensitivity to immune effectors or depleting the tumor microenvironment of Treg cells and MDSCs (72, 73) have been shown to promote intratumor expression of chemokines attracting T cells (74).

Taken together, these findings support the concept that increasing T-cell traffic to the tumor, possibly in association with immunogenic chemotherapy, may be a valid strategy to enhance response to immunotherapy in cancer patients.

## Reprograming Energy Metabolism in Cancer

Reprograming energy metabolism is an emerging hallmark of cancer closely linked to hypoxia and neoangiogenesis (25). Indeed, uncontrolled cell proliferation requires a continuous adjustment of energy metabolism in order to fuel cell growth and division also in the absence of adequate tumor perfusion (14). As early as in 1930, Otto Warburg showed that cancer cells craving for energy take up much more glucose than normal cells and mainly process it through aerobic glycolysis, the so-called “Warburg effect” (75). Curiously enough, also T cells that differentiate from the naïve to the effector state upregulate genes encoding glycolytic enzymes (76), but tumor cells incorporate 10- to 100-fold greater glucose than T cells over a fixed time period (77), thus suggesting a biased competition for glucose between cancer cells and activated T cells within the same microenvironment.

**Figure 3:**
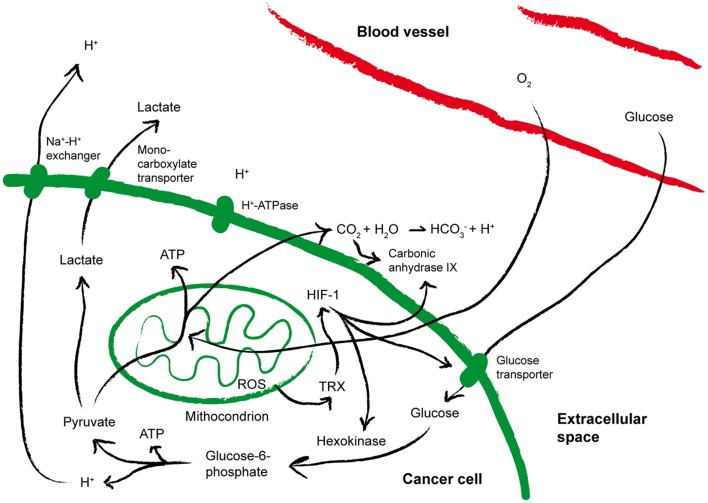
**Metabolic alterations within the tumor microenvironment**. The cartoon summarizes the metabolic alterations often found within the tumor microenvironment that may impact on T cell fitness. See the text for more details. ATP, Adenosine-5′-triphosphate; HIF-1, hypoxia inducible factor 1; ROS, reactive oxygen species; TRX, thioredoxin.

As summarized in Figure 3, a direct consequence of aerobic glycolysis is the production of lactate from pyruvate, and acidic metabolites that cause drop in extracellular pH (78), which may select for more aggressive acid-resistant clones and favor tumor invasion (14). Pyruvate decarboxylation within mitochondria causes the generation and subsequent release of CO_2_, which favors increased expression of carbonic anhydrase IX (CA-IX), a cancer-associated membrane bound isoform of the enzyme carbonic anhydrase that catalyzes the hydration of CO_2_ to bicarbonate and H^+^, thus contributing to acidify the extracellular microenvironment of tumors (79, 80). A low extracellular pH triggers the activation on tumor cell membranes of transporters that protect the cytosol from acidosis. In addition, hypoxia stabilizes the heterodimer HIF-1, which in turn induces the up-regulation of glucose transporters and CA-IX, thereby increasing acidity within the tumor microenvironment (80). As a result, while in normal tissues the extracellular pH is maintained around 7.4, in malignant tumors the pH can drop to values of 6.0 and less, with averages of 0.2–0.6 units lower than in normal tissues (81). The tumor-supporting role of low pH has been recently corroborated by the observation that pharmacologic inhibition of CA-IX or of the vacuolar H^+^-ATPases display antineoplastic effects (79, 82).

Hypoxia and pH are also strongly tangled with reduction-oxidation (redox) reactions (Figure 3). Already at the earliest stages of tumor development, free radicals, HIF-1-induced gene expression and hypoxia are strictly interconnected (83). Indeed, reactive oxygen species (ROS) are generated in mitochondria of cells exposed to low oxygen (84), and the phenomenon is further amplified by cyclic reoxygenation. Also the anti-oxidant systems upregulated by tumor cells to counterbalance oxidative stress contribute to the altered redox of the tumor microenvironment and to tumor progression. Overexpression of reducing enzymes such as thioredoxin (TRX) has been found in many tumors and correlated to poor prognosis (85, 86). TRX induces and stabilizes HIF-1α (87), and co-localizes with both HIF-1α and CA-IX in hypoxic areas of the tumors (79). In addition, proton pumps have been proposed to de-toxify tumor cells from microenvironmental ROS (88). Thus, hypoxia, acidosis, redox-remodeling can cooperate to establish a more aggressive malignant phenotype, and possibly to promote the derangement of immune functions (77).

## Alterations of the Tumor Metabolism That Impact on T Cell Fitness

The immune system has been proposed as sensor of the metabolic state (89). Bidirectional communication and coordination between metabolism and immunity, while effective in maintaining and defending the internal environment from the environment around us, may result in inhibition of immune functions and may favor chronic inflammation and cancer. A well-known example of metabolism-mediated limitation of the function and survival of TILs is tryptophan consumption by tumor cells and antigen presenting cells (APCs) producing IDO (90). This mechanism can also restrain the therapeutic efficacy of checkpoint blockade strategies such as targeting of cytotoxic T lymphocyte antigen-4 (CTLA-4), glucocorticoid-induced TNFR family related gene (GITR), and the PD-1/PD-L1 axis (91).

More specifically, hypoxia, acidosis, and redox-remodeling are all perceived as sensors by the immune system. Thus, as summarized in Table 1, hypoxia inhibits TCR-triggered signaling, proliferation and cytokine production by T cells (92, 93). Intracellular HIF-1α appears to have a direct role in T cell inhibition, since HIF-1α is induced upon TCR triggering (94, 95), it is further increased in hypoxic conditions, and knocking down HIF-1α in T cells increases their cytokine production potential both *in vitro* and *in vivo* (94). T cells are also inhibited by hypoxia-driven accumulation of extracellular adenosine (96).

**Table 1:** **Effects of the tumor metabolism on TILs**.

Metabolic alteration	Species	Inhibition	Promotion	Reference
Hypoxia	*Mus musculus*	Expansion of CD8^+^ T cells	Development of more lytic CTLs	Caldwell et al. (92 )
		IL2 and IFNγ production by CD8^+^ and CD4^+^ T cells	VEGF production	
			Expression of TCR and LFA-1 on CD8^+^ T cells	
	Human	Voltage-dependent K^+^ channels		Conforti et al. (93 )
	*Mus musculus*		HIF-1α expression	Lukashev et al. (94)
	*Mus musculus*		Accumulation of extracellular adenosine	Sitkovsky et al. (96)
	Human and *Mus musculus*		Treg recruitment	Facciabene et al. (97)
	*Mus musculus*	Treg differentiation	Th17 differentiation	Dang et al. (98)
	Human		Th17 survival	Kryczek et al. (100)
	*Mus musculus*	T cell-mediated cytotoxicity		MacDonald (105)
Low intratumor pH	Human and *Mus musculus*		Lymphocyte apoptosis	Lugini et al. (108), Calcinotto et al. (113)
	*Mus musculus*	CTL response *in vivo*	CTL activation *in vitro*	Droge et al. (109)
	*Mus musculus*	IL2-mediated T cell proliferation		Ratner (110)
	*Mus musculus*	CTL-mediated cytotoxicity		Redegeld et al. (111)
	Human	Proliferation and effector function of T cells		Fischer et al. (112)
	Human and *Mus musculus*	CTL proliferation, cytolitic activity and IL2, TNFα and IFNγ production		Calcinotto et al. (49)
Oxidative stress	Human and *Mus musculus*		Down-modulation of TCR CD3ζ chain	Rodriguez et al. (122), Rodriguez et al. (123)
	*Mus musculus*	Activation of JAK, STAT, ERK and AKT		Bingisser et al. (125), Mazzoni et al. (126)
	*Mus musculus*	Conformational flexibility of TCR and CD8 molecules		Nagaraj et al. (134)
	Human and *Mus musculus*	Intratumor infiltration of T cells		Molon et al. (135)
	Human		Release of cysteine and TRX by DCs	Angelini et al. (137)

More recently it has been reported that hypoxia within the tumor microenvironment promotes Treg recruitment through the induction of CC-chemokine ligand 28 (97). Conversely, up-regulation of HIF-1α under hypoxic conditions inhibits Treg differentiation through FoxP3 degradation, and favors the differentiation of Th17 cells by directly inducing RAR-related orphan receptor gamma t (RORγt) transcription (98) and glycolytic genes (99). HIF-1α also induces several survival promoting genes in Th17 cells, thus preventing their apoptosis (100). Th17 cells are a subpopulation of T helper cells producing IL17, IL17F, and IL22, which play a critical role in immunity to certain pathogens and autoimmune inflammation (101). The role of Th17 cells in cancer is more debated. Indeed, Th17 exert anti-tumorigenic activities, likely by facilitating the recruitment of other effector immune cells (102), and pro-tumorigenic activities by inducing tumor vascularization and the release of tumor-promoting factors by tumor and stromal cells (103). Thus, the effects of hypoxia on the tumor microenvironment are rather complex, and the use of HIF inhibitors for therapeutic purposes should be carefully balanced to avoid the dominance of pro-tumorigenic over anti-tumorigenic mechanisms.

Hypoxia may also render the tumor cells more resistant to cytotoxic T lymphocyte (CTL)-mediated lysis through HIF-1α-dependent induction in cancer cells of miR-210, which downregulates the expression of *PTPN1*, *HOXA1*, and *TP53I11* genes (104). It remains to be defined how coordinated silencing of these three genes affects cancer cell susceptibility to CTL lysis. The effect of hypoxia on CTLs is still debated (77). Interestingly, simultaneous glucose deprivation and hypoxia block T cell-mediated cytotoxicity *in vitro* (105), therefore suggesting an additional mechanism of immunosuppression within the tumor microenvironment.

There are relatively few reports on the impact of low intratumor pH on T cells (106). Clinical evidence suggests that metabolic acidosis is often associated with immunodeficiency (107). Indeed, both leukocyte activation and the bactericidal capacity of leukocytes are generally impaired at reduced pH (106) suggesting that T cells could be extremely sensitive to pH variations. Lymphocytes also die at the same acidic pH malignant tumor cells perfectly remain alive (108). Droge et al. (109) studied the effect of lactate on murine T-cell populations and found that lactate is able to suppress the CTL response *in vivo*, whereas activation of CTLs *in vitro* is increased. Few years later, it was reported that the proliferation of IL-2-stimulated T cells is inhibited at pH 6.7 (110), and the cytolytic activity against cancer cells of CTLs is markedly reduced when T cells are exposed to acidic pH (111). More recently, Fischer and colleagues demonstrated that high lactic acid concentrations, as the ones found in the tumor environment, block lactic acid export in human T cells, thereby disturbing proliferation and effector functions (112).

We have found that lowering the pH *in vitro* to values most frequently detected within tumors (pH 6–6.5) induces hyporesponsiveness in both human and mouse tumor-specific CTLs, which is characterized by impaired proliferation, cytolytic activity, and cytokine secretion (113). Interestingly, buffering of culture pH to physiologic values associates with the complete recovery of T cell functions, although longer exposure or lower pH values causes permanent damage and T cell death (113), arguing that a portion of T cell immunity might be lost at tumor site when extreme metabolic alterations are present. From a molecular standpoint, TCR triggering at low pH associates with reduced expression of IL-2Rα (CD25) and TCR, and diminished activation of signal transducer and activator of transcription 5 (STAT5) and extracellular signal-regulated kinase (ERK) (113), signaling alterations frequently found in anergic T cells (114, 115). Interestingly, similar characteristics were found in tumor-specific CTLs infiltrating melanoma lesions, whose pH was 6.5 (113). Thus, acidity *per se* is a novel tumor cell extrinsic mechanism of immune escape (116).

Whereas redox-activated signaling events are physiologically needed both as antimicrobial defense and to guarantee the correct spatial and temporal extension of the immune reaction, redox-remodeling within the tumor microenvironment negatively affects immune surveillance. Indeed, oxygen ions and peroxides are potent antibacterial agents produced by phagocytic cells including macrophages and neutrophils (117). ROS are also implicated in NLRP3 inflammasome activation in myeloid cells (118). It has also been increasingly appreciated that endogenous ROS are required for optimal T cell activation (119). Yet, exogenous oxidative stress may dramatically suppress T cell activation and effector functions. As an example, macrophages within the tumor microenvironment express inducible nitric oxide synthase (iNOS) and can induce tumor killing by generating large amounts of nitric oxide (NO). However, iNOS is also expressed by MDSCs, a heterogeneous population of cells of myeloid origin that include immature macrophages, granulocytes, DCs and other myeloid cells (57). MDSCs also express arginase 1 (Arg1) that together with iNOS metabolizes the essential aminoacid arginine to either l-ornithine and urea, or to l-citrulline and NO (120, 121). Depletion of arginine from the microenvironment induces T cell dysfunction because of loss of CD3ζ chain expression (122, 123), and prevents the up-regulation of cell cycle regulators by these cells (124), thus blocking their proliferation. In addition, NO blocks the activation of Janus-activated kinase 1 (JAK1), JAK3, STAT5, ERK, and AKT (125, 126), thus suppressing several T cell functions (125–129).

Depletion of l-arginine may also trigger superoxide $\left({\text{O}}_{2}^{-}\right)$ generation from iNOS (130, 131), which is eventually converted to hydrogen peroxide. ROS contribute to the MDSC-mediated suppression of tumor-specific T cell responses in tumor-bearing mice (57, 132).

Finally, the reaction between NO and $O_{2}^{-}$ generates reactive nitrogen-oxide species (RNOS), among which peroxynitrite (ONOO^−^) (133). ONOO^−^ mediated nitration of tyrosine residues in the TCR and CD8 co-receptor causes a decreased conformational flexibility of these molecules and failure in proper T cell activation (134). Nitration of chemokines also prevents intratumoral infiltration of antigen-specific T cells (135).

Also Tregs modulate the redox of the microenvironment by subtracting cysteine necessary to effector T cell, function (136). Indeed, DCs within the tumor microenvironment may have additional nutritional and redox-remodeling roles, since they reduce the extracellular microenvironment required for T cell activation by releasing cysteine and TRX (137). In the same vein, Tregs diminish glutathione synthesis in DCs and consume extracellular cysteine (138), thus remodeling extracellular redox.

Additional hypoxia-driven metabolic dysfunctions, leading to the accumulation of extracellular adenosine, further increased by Tregs (139, 140), could act in synergy with acidic pH in dampening T cell function through A2A adenosine receptor-driven cAMP intracellular accumulation (96).

All together these findings sustain the concept that hypoxia, nutrient deprivation, abnormal glycolysis, and low pH act in synergy crippling immune surveillance (Figure 1).

Also alterations of the lipid metabolism that occur in the tumor microenvironment might affect T cell functions [e.g., (141)], but direct *in vivo* evidences of this phenomenon are poor.

## Strategies That Impact on Tumor Metabolism and Promote Fitness of Tumor Infiltrating Lymphocytes

Different therapeutic approaches have been proposed to modulate hypoxia, tumor acidity or redox, which directly or indirectly affect TIL viability and effector functions (Figure 1). Being the tumor microenvironment so complex and redundant, the risk remains that interfering with one metabolic pathway, thus inhibiting one pro-tumoral mechanism, may favor another. For the sake of brevity, we will touch upon some clarifying examples.

It has been reported that the anti-VEGF monoclonal antibody bevacizumab induces intratumoral hypoxia, likely through excessive vessel remodeling (142), thus increasing the population of cancer stem cells (CSCs) in human breast cancer xenografts (143), and promoting epithelial to mesenchymal transition (EMT) in a murine model of bevacizumab-resistant pancreatic cancer (144). Angiogenesis inhibitors targeting the VEGF pathway may also elicit tumor adaptation and progression to stages of greater malignancy, with heightened invasiveness and in some cases increased lymphatic and distant metastasis (145). Bevacizumab has also been shown to induce malignant traits through induction of paracrine factors, which recruited pro-angiogenic myeloid cells (146), whose phenotype is reminiscent of MDSCs. Thus, anti-angiogenic compounds while cutting nutritional support to tumor cells, may favor local hypoxia and MDSC accumulation.

Also sunitinib, a receptor tyrosine kinase inhibitor with anti-angiogenic properties (147), which has been recently approved for the treatment of metastatic renal cell carcinoma (148), induces hypoxia, through a yet undefined mechanism, and increase in CSCs (143). Interestingly, semaphoring 3A (Sema3A), an endogenous anti-angiogenic agent, counteracts sunitinib-induced tumor hypoxia, and Sema3A and sunitinib synergize to enhance survival of tumor-bearing mice (149). In addition, one cycle of treatment with sunitinib is sufficient to increase the proportion of type 1 T cells (150), likely by reducing MDSCs (151). These findings have been confirmed in mouse models of cancer, in which sunitinib reduced viability and proliferation of MDSCs (152) and their accumulation in tumors (153).

Several drugs have been identified that target HIF-1α, thus inhibiting angiogenesis (154). However, HIF-1 inhibitors may impact on balance between Treg and Th17 cells favoring the former (99, 155). Thus, further investigation is needed to fully elucidate the therapeutic potential of HIF-1 inhibitors in cancer patients.

Conversely, hypoxia can be skillfully utilized to selectively target TILs. Indeed, hypoxia induces expression of CD137 (4-1BB) on TILs, and low-dose intratumoral injections of agonist anti-CD137 monoclonal antibodies avoid systemic toxicity while achieving anti-tumor systemic effects (156). In addition, intratumoral anti-CD137 antibodies synergized with systemic blockade of PD-L1 (156).

Several strategies have been proposed to neutralize intratumor acidity and therefore affect TILs. Robey et al. (157) reported that oral treatment with NaHCO_3_ increased the extracellular pH of spontaneous metastases, inhibited cancer cell extravasation and colonization in mouse models of breast and prostate cancer. However, no information is available on the effects of systemic administration of bicarbonate on T cells and there is some concern related to the risk of metabolic alkalosis.

We obtained evidence that systemic administration of the PPI esomeprazole (12.5 mg/Kg) to tumor-bearing mice caused a rapid (within 60 min) increase in tumor pH, which associated with enhanced IFNγ production by TILs (113). Indeed, on a per cell basis, TILs in the tumor of PPI-treated mice produced more IFNγ than TILs from mice treated with vehicle (113). PPI treatment also increased phosphorylated ERK in TILs, thus giving molecular support to the PPI-mediated effect. As expected for a drug that is administered as a pro-drug and requires protonation at low pH (158), IFNγ production by T cells isolated from the spleen, lung, and kidney of mice treated either with PPIs or vehicle did not differ (113), thus suggesting that also the effects of PPIs on T cells are restricted to area of acidosis. PPIs also affected adoptively transferred T cells that reached the tumor, and PPI treatment increased the therapeutic potential of both active and adoptive immunotherapies (113). Because of the high selectivity for an acidic milieu and instability, PPIs can be safely used at high doses (158) as the one tested by us (113), which also affect tumor cells *in vivo* (159). Thus, PPI treatment may represent a promising strategy for recovering specific immunity and improving the efficacy of T cell-based cancer treatments.

PPIs are also known for their anti-oxidant and anti-inflammatory activities (160). Vacuolar proton pumps are expressed in the membrane of phagolysosomes of neutrophils, and lysosomal acidification is relevant for neutrophil oxidative burst. Thus, PPIs reduce release of ROS by neutrophils further impacting on the tumor microenvironment. Whiles the mechanism of action of PPIs on leukocytes is still under investigation (160), our data suggest that *in vivo* PPIs enhance anti-tumor activities of TILs (113, 116).

Cancer cells promote chronic autophagy as survival adaptation to the acidic microenvironment (161). Because at least *in vitro* autophagy can also be induced in tumor cells by PPIs (162), strategies might be devised to inhibit autophagy during PPI treatment, yet taking into account the potentially negative effects of autophagy inhibitors on TILs (163).

Regarding redox, it has been reported that the anti-diabetic drug metformin or the mTOR inhibitor rapamycin, restore catabolic mitochondrial fatty acid oxidation and favor the induction of memory T cells, thus increasing the therapeutic efficacy of cancer vaccines (141, 164, 165).

Several pre-clinical studies also support the use of A2A adenosine receptors (A2ARs) antagonists to increase T cell activity within the tumor microenvironment (166). As an example, the compounds ZM241385 or 1,3,7-trimethylxanthine (caffeine) showed to increase the anti-tumor activity of adoptively transferred T cells in mice bearing large tumors (167). Curiously, drinking coffee was found to correlate with significant decreased risk of cutaneous malignant melanoma only in women (167, 168), suggesting that caffeine may also impact on cancer immune surveillance.

Finally, therapeutic strategies targeting either Tregs (169, 170) or MDSCs (171, 172); (173, 174), collaborate in making the tumor microenvironment more permissive for TIL survival and anti-tumor activities. Interestingly, MDSCs impair T cell trafficking through down-regulation of CD62L on CD4 and CD8 T cells (175) and chemokine nitration (135). Thus, therapeutic strategies that block MDSCs accrual at the tumor site and their immunosuppressive function, and more specifically drugs interfering with chemokine nitration, are expected to significantly improve the efficacy of both active and adoptive immunotherapies.

## Conclusion and Perspectives

Despite considerable progress over the last decade, the tumor microenvironment is an area of research that remains ripe for further investigation, especially with regard to the relentless and dynamic modifications in its cellular composition and metabolism.

Accumulating experimental evidence lends weight to the concept that the most effective therapeutic strategies against cancer will be the ones that consider the tumor and its microenvironment as a whole, and yet simultaneously and coordinately address several individual aspects of this complex system. So far, either chemotherapy, surgery or radiotherapy have been combined with either one of active immunotherapy and/or adoptive T cell therapy, checkpoint blockade strategies or drugs that modify the vascularization and metabolism of the tumor (38, 50, 116, 176–178), thus improving distribution and synergistic anti-cancer activity of drugs and T cells. A step forward will be to carefully devise multiple targeted therapies that simultaneously or subsequently attack tumor cells and the diverse aspects of the tumor microenvironment, and yet preserve the function of organs not involved by the neoplasm. Thus, it can be anticipated that adoptive and active immunotherapy given together with treatments that transiently normalize and/or activate tumor-associated ECs and drugs that impact on tumor metabolism and reduce the local immunosuppressive environment would greatly benefit cancer patients without causing relevant systemic toxic effects. As an example, in TRAMP mice affected by advanced prostate cancer, the combination of non-myeloablative total body irradiation, hematopoietic stem cell transplantation, infusion of donor mature lymphocytes, and tumor-specific vaccination overcomes tumor-specific T cell tolerance, prompts tumor debulking, and induces long-lasting tumor-specific memory response that protects mice from tumor recurrence (70). Interestingly, the addition of NGR-TNF at the peak of the vaccination-induced immune response favors penetration of activated T cells within the transformed prostate epithelium (49) and guarantees an even stronger anti-tumor activity (Mondino A. Personal communication). We are investigating the possibility to add PPIs to this already complex combined therapy to favor the anti-tumor activity of adoptively transferred and vaccine-induced T cells that have reached the prostate.

Given the outstanding results obtained with immune checkpoint blockers in cancer patients (179), it will be particularly interesting to investigate the therapeutic efficacy of their combination with metabolism and vessel modulators.

It is also important to underline that more is not always better (180). One example is the recent failure of a well-designed and carefully analyzed multi-institutional clinical trial in which 732 patients with previously untreated metastatic colorectal cancer were randomly assigned to receive the combination of capecitabine, oxaliplatin, and bevacizumab (i.e., a monoclonal antibody against VEGF) or the same three drugs with cetuximab (i.e., a monoclonal antibody directed against the epidermal growth factor receptor; EGFR) (181). The four-drug combination resulted in significantly shorter progression-free survival and inferior quality of life. A similarly negative effect was obtained when another anti-EGFR monoclonal antibody (i.e., panitumumab) was added to the combination of folinic acid, fluorouracil, oxaliplatin, and bevacizumab in previously untreated metastatic colorectal cancer patients (182).

Thus, additional investigation is needed to define the best settings for each combination approach. In this perspective, reliable animal models of human diseases remain instrumental.

## Conflict of Interest Statement

The authors declare that the research was conducted in the absence of any commercial or financial relationships that could be construed as a potential conflict of interest.
